# *Plasmodium falciparum* Expressing Domain Cassette 5 Type PfEMP1 (DC5-PfEMP1) Bind PECAM1

**DOI:** 10.1371/journal.pone.0069117

**Published:** 2013-07-09

**Authors:** Sanne S. Berger, Louise Turner, Christian W. Wang, Jens E. V. Petersen, Maria Kraft, John P. A. Lusingu, Bruno Mmbando, Andrea M. Marquard, Dominique B. A. C. Bengtsson, Lars Hviid, Morten A. Nielsen, Thor G. Theander, Thomas Lavstsen

**Affiliations:** 1 Centre for Medical Parasitology at Department of International Health, Immunology and Microbiology, Department of Infectious Diseases, Copenhagen University Hospital (Rigshospitalet), University of Copenhagen, Copenhagen, Denmark; 2 National Institute for Medical Research (NIMR), Tanga Medical Research Centre, Tanga, Tanzania; Bernhard Nocht Institute for Tropical Medicine, Germany

## Abstract

Members of the *Plasmodium falciparum* Erythrocyte Membrane protein 1 (PfEMP1) family expressed on the surface of malaria-infected erythrocytes mediate binding of the parasite to different receptors on the vascular lining. This process drives pathologies, and severe childhood malaria has been associated with the expression of particular subsets of PfEMP1 molecules. PfEMP1 are grouped into subtypes based on upstream sequences and the presence of semi-conserved PfEMP1 domain compositions named domain cassettes (DCs). Earlier studies have indicated that DC5-containing PfEMP1 (DC5-PfEMP1) are more likely to be expressed in children with severe malaria disease than in children with uncomplicated malaria, but these PfEMP1 subtypes only dominate in a relatively small proportion of the children with severe disease. In this study, we have characterised the genomic sequence characteristic for DC5, and show that two genetically different parasite lines expressing DC5-PfEMP1 bind PECAM1, and that anti-DC5-specific antibodies inhibit binding of DC5-PfEMP1-expressing parasites to transformed human bone marrow endothelial cells (TrHBMEC). We also show that antibodies against each of the four domains characteristic for DC5 react with native PfEMP1 expressed on the surface of infected erythrocytes, and that some of these antibodies are cross-reactive between the two DC5-containing PfEMP1 molecules tested. Finally, we confirm that anti-DC5 antibodies are acquired early in life by individuals living in malaria endemic areas, that individuals having high levels of these antibodies are less likely to develop febrile malaria episodes and that the antibody levels correlate positively with hemoglobin levels.

## Introduction

WHO has estimated annual malaria mortality to around 655.000, but this number has been challenged by a recent study estimating malaria mortality to around 1.240.000 [1,2]. *Plasmodium falciparum* is the most pathogenic malaria parasite species infecting humans. The pathogenicity of *P. falciparum* is related to expression of *P. falciparum* Erythrocyte Membrane Protein 1 (PfEMP1), a variable surface antigen encoded by the *var* gene family [3–5]. PfEMP1 exposed on the surface of erythrocytes infected with late-stage parasites mediate their sequestration in deep vascular beds by adhering to host cell receptors expressed on microvascular endothelial cells, such as CSA, ICAM1, PECAM1 and CD36 [6–10]. Sequestration protects the parasite from splenic clearance, and thereby confers a selective advantage. Sequestration can lead to microvascular obstruction, acidosis and inflammation in the capillaries and together with high parasite burden may cause severe complications such as cerebral malaria, respiratory distress or severe malarial anemia [11].

Parasites causing severe malaria are thought to express PfEMP1 that are superior in their ability to sequester due to particularly high binding affinities to their endothelial cell ligands, which causes higher effective *in vivo* multiplication rates. Parasites expressing such PfEMP1 are thought to dominate infections early in life where immunity to these variants has not yet been acquired [12]. This would explain why individuals in areas with intense transmission experience severe malaria symptoms during childhood, but continue to harbour parasites causing uncomplicated disease as they become adults. In these areas, natural immunity towards severe malaria is acquired at a young age, and it appears that only a few disease episodes are required to acquire protection from severe malaria [13]. A large body of evidence has shown that infections causing severe malaria in children are linked to the expression of a restricted subset of PfEMP1 and that protective antibody-mediated immunity is acquired to these variants [14–27].

Each *P. falciparum* genome harbours ~ 60 different PfEMP1-encoding *var* genes encoding large (250-350 kDa) proteins composed of two to nine Duffy Binding Like (DBL) and Cysteine-rich InterDomain Region (CIDR) domains. Based on the orientation of their upstream sequences (UPS) and the structure of the N-terminal DBL-CIDR domain shared by most PfEMP1, *var* genes are divided into three major groups, A, B and C [28], and conserved unique variants called VAR1, VAR2CSA and VAR3. In addition, 21 conserved PfEMP1 domain compositions named domain cassettes (DC) have been identified [29]. So far, only one receptor: ligand pair, the binding of VAR2CSA to Chondroitin Sulfate A (CSA) [30] has been unambiguously associated with a particular malaria complication: the sequestration of parasites in the placenta leading to severe malaria in pregnant women [31,32]. However, the structured PfEMP1 repertoire suggests that separate DC types confer specific receptor binding phenotypes on infected erythrocytes [29].

Several studies have pointed to group A and B/A PfEMP1 as being associated with severe malaria in children [19,20,23,25–27,33–36], but until recently, field studies have not been helpful in defining specific DC types of particular clinical relevance and the binding phenotype they conveyed. Parasites expressing DC5 (var5), DC8 and DC13 variants have been associated with severe malaria in Tanzanian children. In three independent studies, panning on endothelial cells selected for parasites expressing DC5, DC8 and/or DC13, demonstrating particularly efficient endothelial cell adhesion of these variants [20,37,38]. We have previously shown that antibody levels to a 3D7 group A *var* gene encoded PfEMP1 (PF11_0008) containing DC5, are associated with protection from malaria fever and that these antibodies are acquired in early childhood or following a single experimental infection [19,39,40].

In this study, we analysed whether levels of antibodies specific for the DC5 in PF11_0008 (3D7) associate with protection from different malaria complications. In addition, we employed human endothelial cells to determine the adhesion phenotype of DC5-PfEMP1-expressing infected erythrocytes. Finally, we elicited anti-PfEMP1 antibodies in rats in order to demonstrate the possibility of inducing cross-reactive antibodies to DC5-PfEMP1 variants.

## Materials and Methods

### Ethics statement

All experiments including immunizations and bleeding of animals was approved by The Danish Animal Procedures Committee (‘‘Dyreforsoegstilsynet’’) as described in permit no. 2008/561-1498 and according to the guidelines described in act no. LBK 1306 (23/11/2007) and BEK 1273 (12/12/2005). Written informed consent was obtained from all individuals studied. From child participants, written informed consent was from a parent or the guardian. Ethical clearance was granted by the Ministry of Health and the Ethics Committee of the National Institute for Medical Research in Tanzania (NIMR/HQ/R.8a/Vol.IX/559).

### Sequence analysis

PfEMP1 amino acid sequences were aligned using MUSCLE [41], viewed using BioEdit [42] and Neighbour Joining distance trees were built using MEGA [43]. The Poisson correction model was used with gaps/missing data treated as pairwise deletion. Bootstrap proportions were computed after 1000 replications to assure confidence in topology. Sequence LOGOs were generated from protein alignments using WebLogo [44].

### Malaria parasite culture

Malaria parasite lines were cultured according to standard procedures [45]. Briefly, parasites were maintained in type O^+^ erythrocytes at a hematocrit of 5% in RPMI-1640 HEPES medium supplemented with 25 mmol/l sodium bicarbonate (Lonza), 2 mmol/l L-glutamine (Sigma-Aldrich), 0.125 µg/ml gentamycin (Lonza), and 0.125 µg/ml Albumax II (Invitrogen). Culture bottles with infected erythrocytes were gassed with a mixture of 2% O_2_ and 5% CO_2_ in nitrogen and placed in a 37°C incubator. Parasite cultures were analysed routinely for Mycoplasma infection using the MycoAlert kit (Lonza), and the genetic identity of the parasite lines was regularly verified by PCR at the polymorphic *msp2* and *glurp* loci.

### Culture of TrHBMEC

Transformed human bone marrow endothelial cells (TrHBMEC) have been found not to express CD36 but express PECAM1 and ICAM1 constitutively, although baseline ICAM1 expression (before TNF-α, IL-1β or IFN-γ stimulation) is low [46]. The cells were cultured in 500 ml DMEM (Lonza) supplemented with 50 ml fetal calf serum (FCS) (Lonza), 5 ml RPMI vitamin (Sigma-Aldrich), 50000 U pen/strep (Lonza) and 2 mmol/l L-glutamine. Filtercap culture flasks were preincubated with 2 ml 0.2% gelatin in water for 30 min. at 37°C before adding cells to the flasks. Cells were grown at 37°C with 5% CO_2_ in water-saturated air.

### *In vitro* selection of parasites

Rat serum was depleted for anti-human erythrocyte receptor antibodies by 1:1 incubation of rat serum and type O^+^ erythrocytes over night at 4°C, followed by removal of erythrocytes. 3D7 and FCR3 were selected with depleted rat antisera raised against the DBLδ5-CIDRβ4 domains of PF11_0008 or IT4var02, respectively. Selection was performed as previously described [47]. Briefly, infected erythrocytes were incubated with 100 µl rat serum for 1 hour at 37°C, followed by a washing step and a second incubation with biotin-conjugated goat anti-rat IgG (B7139, Sigma). Infected erythrocytes were washed, incubated with streptavidin-coated DynaBeads (M-280, Invitrogen) and bound cells were isolated using magnetic force.

### cDNA synthesis and quantitative Real-time PCR

Erythrocytes infected with late trophozoite and schizont stages were isolated using Magnetic Cell Sorting (MACS, Miltenyi Biotec) [48], allowed to mature and reinvade overnight, and total RNA from ring stage parasites was isolated using Trizol (InVitrogen) as recommended by the manufacturer. Total RNA was treated with DNAse I (Sigma) to digest genomic DNA, and cDNA was reverse transcribed from random hexamers, using Superscript II (Invitrogen) following the manufacturer’s protocols. Quantitative PCR was performed on a Rotorgene 6000 (Corbett Research) in 20 µl reactions using Quantitec SYBR Green PCR master mix (Qiagen) and primer pairs specific for each of the FCR3 *var* genes and internal control genes *seryl-tRNA synthetase* (PF07_0073) and *aldolase* (PF14_0425) as previously described [21,49–51]. Transcript abundance was calculated from primer-specific standard curves and given relative to 1000x the averaged abundance of the two control genes.

### Generation of proteins and antibodies

Recombinant proteins were produced as described previously [19,52,53]. Primer pairs designed to contain restriction enzyme sites (given without restriction sites in Table S1) were used to amplify DBL-encoding fragments from 3D7 genomic DNA. The digested PCR products were cloned into the Baculovirus vector, pAcGP67-A (BD Bioscience), which is designed to contain the V5 epitope upstream of a histidine tag in the C-terminal end of the construct. The identities of the cloned fragments were verified by sequencing. Linearized Bakpak6 Baculovirus DNA (BD Biosciences Clontech) was co-transfected with pAcGP67-A into Sf9 insect cells for generation of recombinant virus particles and histidine-tagged proteins secreted into the supernatant of infected High-Five insect cells were purified on Co^2+^ metal-chelate agarose columns. Eluted products were dialysed overnight in PBS and verified by SDS-PAGE and western blotting using anti-V5 antibodies (Figure S3). Rat antibodies for each protein domain were raised and tested in ELISA for reactivity against the immunizing antigen. All rat antibodies showed high reactivity to their respective domains.

### Adhesion assays

Ring-stage parasites were radioactively labeled by incubation with tritiated hypoxanthine (^3^H) (Amersham; 8.75 MBq/ml packed erythrocytes) in Albumax medium overnight. Flat-bottomed 96-well plates were pre-coated with 120 µl 1% gelatin in water for 30 min./37°C, and 100 µl TrHBMEC cell suspension (450000 cells/ml) was added per well and grown to a monolayer overnight. The following day, parasites were synchronized to the late trophozoite and schizont stage using MACS and adjusted to 1×10^7^ cells/ml in 2% FCS (in RPMI), which was the medium used for the entire assay. Infected erythrocytes were preincubated for 1½ hours/37°C with PBS alone or with anti-DC5-PfEMP1 or control anti-IT4var13 antibodies (IgG-purified in PBS) at an IgG concentration of 0.5 mg/ml or 0.7 mg/ml. Infected erythrocytes were washed to remove surplus IgG. The plated TrHBMEC were washed twice and 25 µl medium was added per well. Antibodies to PECAM1 (BBA7, R&D Systems) and ICAM1 (MCA1615EL, AbDSerotec) were added to the cells at a final concentration of 0.02 mg/ml. Twenty-five µl/well of the preincubated infected erythrocytes was added in triplicates and coincubated with the TrHBMEC on a rocking table for 1½ hours/37°C. Unbound infected erythrocytes were removed with a washing robot (Biomek 2000, Beckman Coulter), the radioactive material harvested (Filtermate Harvester, PerkinElmer), 50 µl/well scintillation liquid (Microscint 20, PerkinElmer) added and radioactivity measured in counts/minute (CPM) (Topcount NXT, PerkinElmer). Adhesion to gelatin-coated wells was also measured in addition to the total amount of radioactivity added per well (max-value). Adhesion was calculated as the proportion (%) of radioactivity in the bound parasites out of the total amount of radioactivity added per well.

### Flow cytometry

Parasite cultures were enriched for late trophozoite and schizont-stage parasites using MACS, adjusted to 2×10^6^ cells/ml in PBS with 2% FCS (PBS2) and 100 µl parasite suspension was added/well to either rat serum or human serum in 96-well plates (NUNC, round-bottomed).

#### Rat serum

IgG was purified from erythrocyte-depleted rat serum as previously described [53]. Twenty µl rat IgG adjusted to 1 mg/ml were incubated with 2×10^5^ infected erythrocytes for 30 min./4°C, washed three times in PBS2, followed by incubation, 30 min./4°C with FITC-conjugated goat anti-rat IgG (62-9511, Invitrogen) diluted 1:150 in PBS2 with 20 µl ethidium bromide/ml (0.1 mg/ml in PBS) (Bie & Berntsen).

#### Human serum

A panel of 69 human serum samples was added to a 96-well plate (5 µl/well). Eight samples were from Danish unexposed controls and 61 samples were from individuals (aged 1-18 years) living in a malaria holo-endemic area (Mgome, Tanzania). Duplicates without serum were also included in the assay. Human serum was incubated with 2×10^5^ infected erythrocytes for 30 min./4°C. Plates were washed in PBS2 three times and stained for 30 min./4°C with 100 µl Goat F(ab')2 Fragment Anti-Human IgG (Fcγ)-FITC (PN IM1627, Ramcon) diluted 1:100 in PBS2 with 20 µl ethidium bromide/ml (0.1 mg/ml in PBS).

After the final incubation, samples were washed twice, resuspended in 100 µl PBS2 and analysed in a flow cytometer (Cytomics FC500MPL, Beckman Coulter). WinList (Verity Software House) was used for analysis of flow cytometry data.

### Confocal Microscopy

Laser scanning confocal microscopy was performed on all samples analysed by flow cytometry. The parasites were synchronized to late trophozoite and schizont stages using MACS and prepared for microscopy as follows. 3D7 PF11_0008, FCR3 IT4var02 and 3D7 PFD1235w parasite lines were incubated with individual rat antibodies against double-domains DBLδ5-CIDRβ4 of PF11_0008 (3D7) and DBLδ5-CIDRβ4 of IT4var02 (FCR3) in separate experiments. Briefly, 1 µl packed infected erythrocytes were washed in 1% BSA in PBS (Sigma). The pellet was incubated in 100 µl BSA/PBS and 3 µl of the respective antibodies for 30 minutes at 4°C. The infected erythrocytes were washed three times in BSA/PBS. The secondary antibody Alexa™488 anti-rat IgG (Invitrogen) (1/2000) and DAPI (300 ng/ml) were added and the mixture was incubated for 30 minutes at 4°C. The infected erythrocytes were washed three times and visualised as live, unfixed cells using a Nikon TE 2000-E confocal Nikon microscope with 60x (numerical aperture 1.4) Apoplan oil immersion objective lens equipped with a Differential Interference Contrast (DIC) system. The images were captured using the frame lambda channels (450/34 and 515/30) and the EZ-Gold imaging system. The images were finally processed using Adobe Photoshop software and displayed with the 5 µm scale bar calculated by the EZ-C1 software.

### Luminex assay

Levels of antibodies towards PfEMP1 in plasma samples were measured with the previously described Luminex assay [19]. In brief, carboxylated Luminex microspheres were covalently coated with the different PfEMP1 protein domains through an interaction of their carboxyl groups and the amino groups on the proteins. Equal volumes of the coated microspheres were pooled together and plasma samples were incubated with the microspheres, followed by sequential addition of biotinylated human IgG (Sigma) detection antibody and streptavidin-phycoerythrin (Sigma). The microspheres were then analysed on a Luminex instrument. The reader was set to read a minimum of 100 microspheres per microsphere region and results were read as median fluorescent intensity (MFI) The MFI values were then translated into arbitrary units based on standard curves obtained by titration of a plasma pool with high levels of anti-PfEMP1 antibodies.

### Analysis of relation between Anti-PfEMP1 antibody level and clinical outcomes

Plasma samples were obtained during cross-sectional village surveys in Mkokola and Kwamasimba as part of ongoing monitoring of malaria endemicity [54]. Anti-PfEMP1 antibody levels were measured in samples collected in April 2004 and association between these levels and risk of having a malaria fever [55] was tested by logistic regression. Malaria was diagnosed in children who reported to health workers between April 2004 and September 2004 with complaints of fever and with a positive malaria blood slide reading. Children with one or more episodes of malaria during the follow-up period were entered into the models as (1), children without a malaria diagnosis in the follow-up period were coded as (0).

Blood hemoglobin levels were measured in April and September 2004. Associations between the hemoglobin levels measured in April or September, respectively, and antibody levels measured in samples collected in April 2004 were analysed by linear regression. Both logistic and linear regression models were corrected for age (using the square root of age in years), use of impregnated bed net, gender and village of residence. Identical statistical models were used for each antigen tested.

## Results

### Conserved sequence traits of DC5-PfEMP1

**Figure 1:**
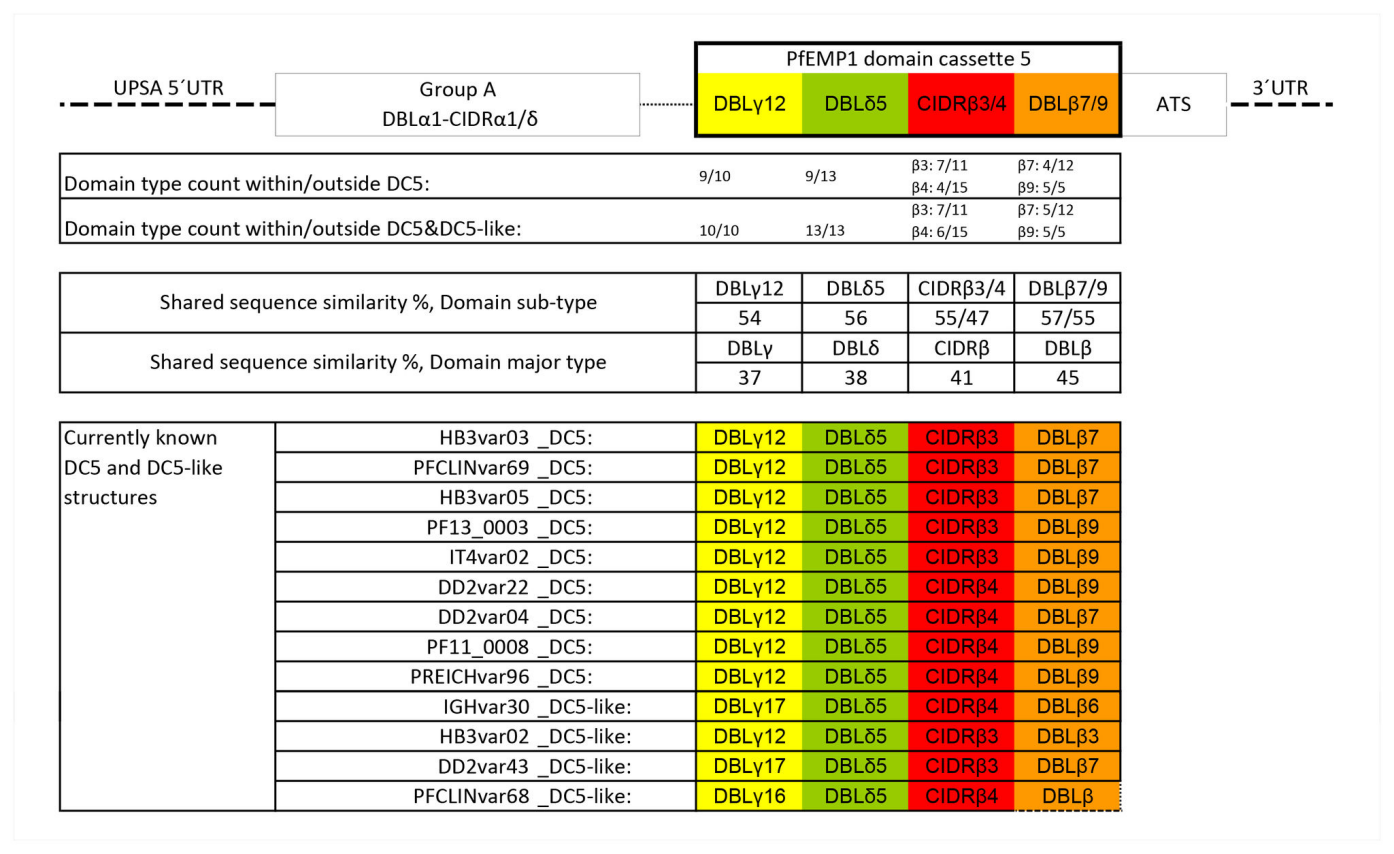
Schematic presentation of known PfEMP1 domain cassette 5. The four-domain DC5 is only found in PfEMP1 molecules of group A. The association of the specific domain type to the DC5 is listed as the count of said domain types found in the DC5 context compared to the total count of the domain type in 399 PfEMP1 sequences from seven near-complete assembled *P. falciparum* genomes [29]. Percentage shared amino acids between domains of the cassette is shown in comparison to percentage shared amino acids of all domains of the major domain class. Lower panel shows domain architecture within DC5 and DC5-like sequences. Dotted lines indicate that only a partial sequence was available for the domain.

The domain cassette 5 (DC5)-PfEMP1 was previously identified as a C-terminal four-domain cassette found in nine group A PfEMP1 sequences present in six of the seven near-complete sequenced *P. falciparum* genomes as well as in the sequenced genome of *P* *. reichenowi* [29]. The four phylogenetically distinct domains forming the cassette (DBLγ12-DBLδ5-CIDRβ3/4-DBLβ7/9) are all predominantly found in the context of the DC5 structure (Figure 1), but it is possible within the seven fully sequenced genomes to identify another four PfEMP1 with domain compositions closely related to DC5, all having the unique C-terminal structure DBLγ-DBLδ-CIDRβ-DBLβ in common. The shared sequence identity of each domain in the structure is shown in Figure 1.

**Figure 2:**
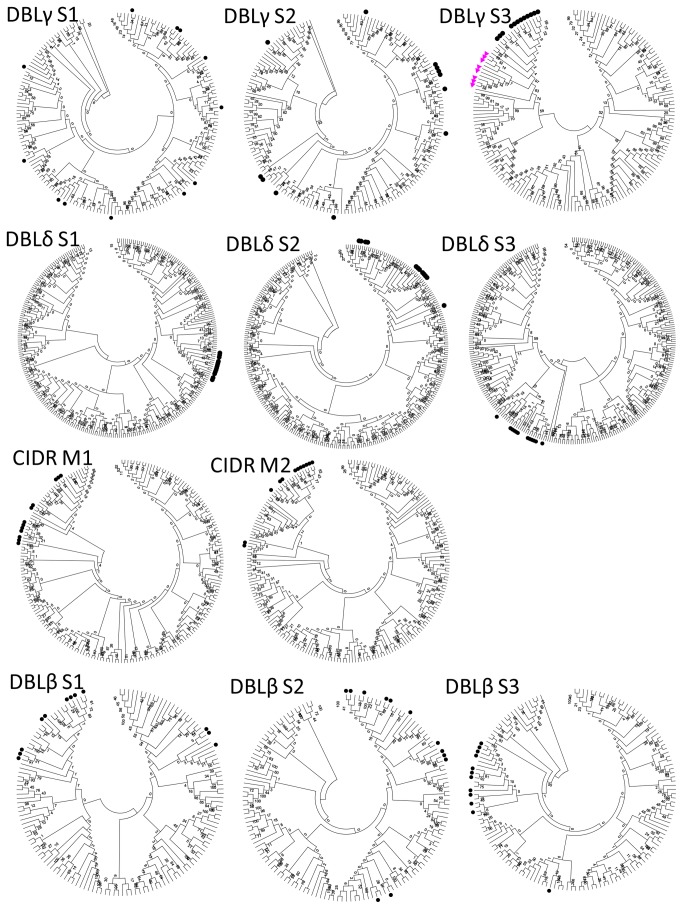
Distance trees of DBLγ, δ, β subdomains S1-3 and CIDR M and M1-2 (p-distance/Neighbour Joining method). Black dots indicate sub-domains found in DC5 sequences. Pink triangles indicate DBLγ S3 domains of DC8 sequences. Numbers at the nodes represent bootstrap proportions on 1000 replicates. DBLγ S3 subdomains of DC5 and 8 as well as all DC5 DBLδ S1 sub-domains form bootstrap supported clusters indicating that these regions are the most characteristic of DC5.

To further define sequence elements characteristic for DC5 and DC5-like domain cassettes, neighbour-joining sequence distance trees were constructed from separate amino acid alignments of DBLγ, δ, β and CIDRβ subdomains (Figure 2). This analysis showed that DBLγ S3 of DC5 and DC5-like sequences form a distinct and bootstrap-supported cluster together with DBLγ S3 of DBLγ6 from DC8-PfEMP1. DC5 and DC5-like sequences also form distinct bootstrapped clusters in the DBLδ S1 tree. In DBLγ S1 and S2, DC5 sequences appear near randomly placed between other DBLγ sequences, whereas DC5 and DC5-like sequences are found closely related, but not in bootstrap supported clusters in the trees of the remaining sub-domain types. In the analysis by Rask et al. 2010 [29], PfEMP1 sequence diversity was also described by 628 unique homology blocks (HB). Some of these are common to all DBL or CIDR domains whereas others are specific for DBL and CIDR domain types. Two homology blocks are only present in DC5 and DC5-like sequences: HB585, found in DBLδ5, and HB331 found in DBLβ7/9. Figure S1 shows a domain annotated sequence LOGO of DC5-PfEMP1, highlighting the positions of HB585 and HB331.

### Antibody recognition of native DC5-PfEMP1 expressing parasites

**Figure 3:**
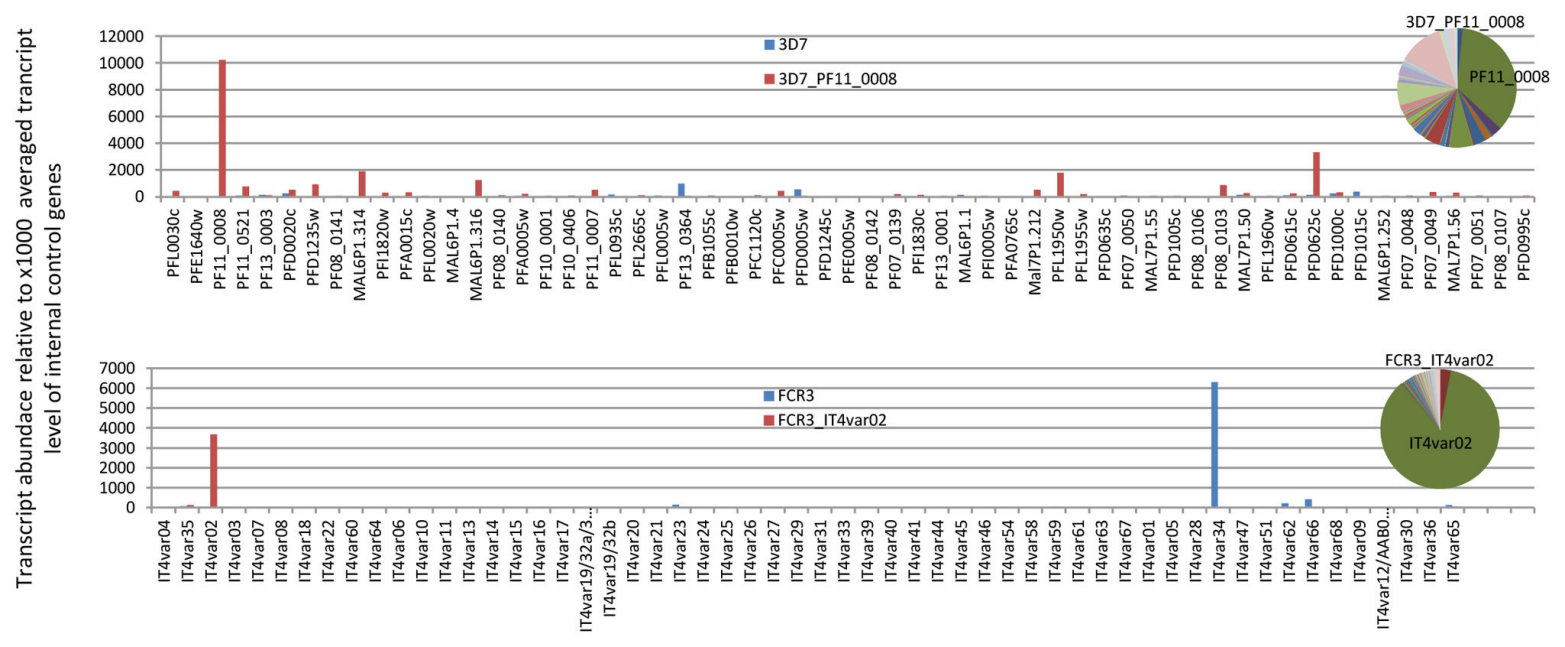
*Var* transcript profiles of 3D7 and FCR3 parasites selected for DC5-PfEMP1 expression. Bars show *var* transcript abundances in 3D7 and FCR3 lines before (blue bars) and after (red bars) selection with DC5 antibodies specific for PF11_0008 (3D7) and IT4var02 (FCR3), respectively. Copy numbers (y axis) are calculated from qPCRs using primers and standard curves specific for each gene, and given relative to 1000 copies of internal *seryl-tRNA synthetase* and *aldolase* controls. Pie charts show the *var* transcript distribution in the selected lines.

**Figure 4:**
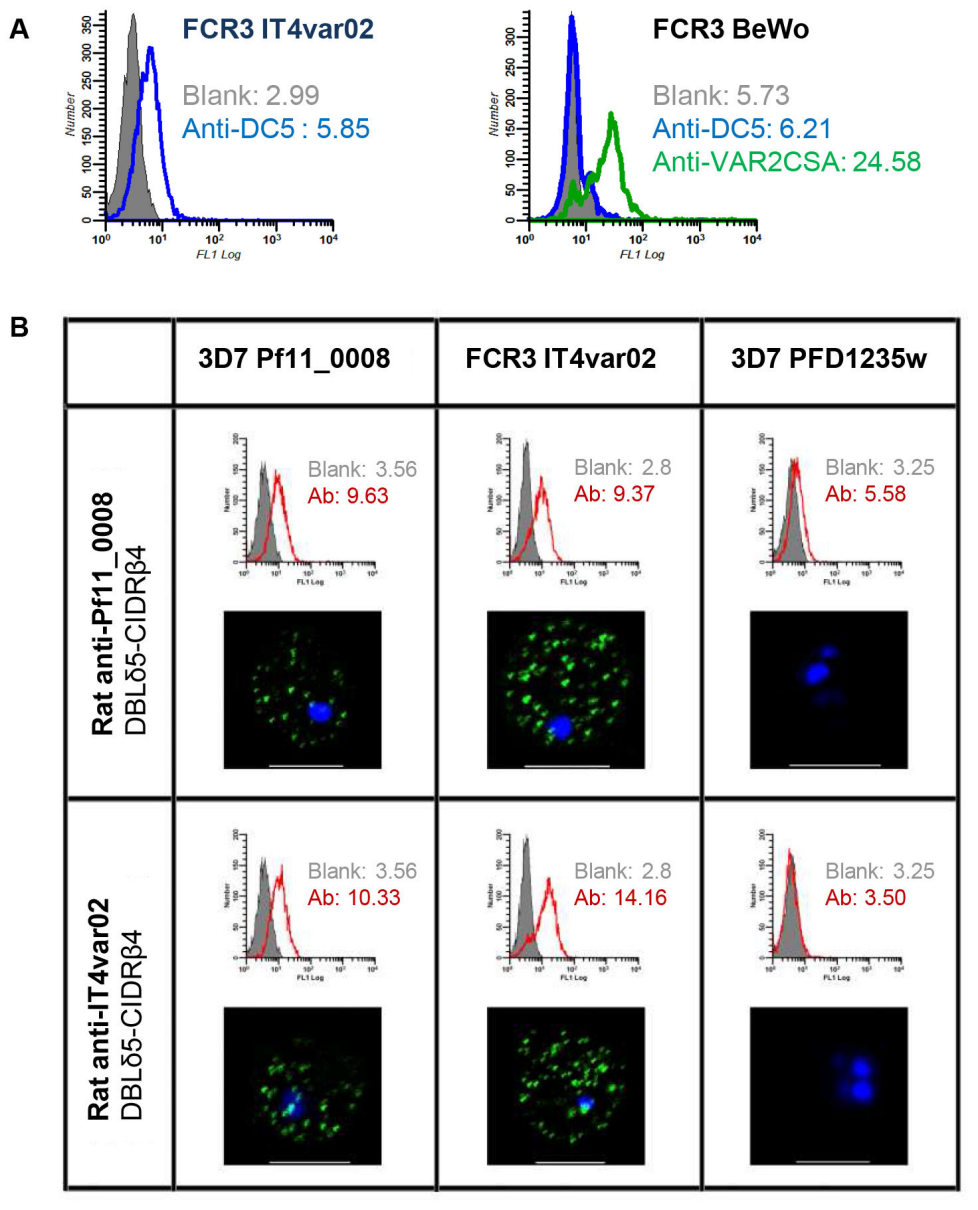
Antibody cross-reactivity to DC5-PfEMP1-expressing 3D7 and FCR3, shown using flow cytometry and confocal microscopy. (A) Flow cytometry histograms showing the reactivity of a *P. falciparum* line expressing IT4var02 containing DC5 and a parasite line expressing VAR2CSA (FCR3 BeWo) to antibodies elicited to the FCR3-DC5 cassette (DBLγ12-DBLδ5-CIDRβ4-DBLβ9) or antibodies against VAR2CSA. (B) Flow cytometry histograms showing the reactivity of parasite lines expressing 3D7 PF11_0008 (3D7-DC5), FCR3 IT4var02 (FCR3-DC5) and 3D7 PFD1235w (3D7-DC4) with antibodies against domains DBLδ5-CIDRβ4 from 3D7-DC5 or FCR3-DC5. Below the flow cytometry histograms are confocal microscopy pictures of the same infected erythrocytes. Surface reactivity with FITC-labeled rat antibodies is seen as green dots and the DNA in the nuclei is stained blue by DAPI.

We expressed the two central DC5 domains (DBLδ5-CIDRβ4) from 3D7 (PF11_0008) and (IT4var02) as recombinant proteins and elicited antibodies against them in rats. The antibodies were used to select DC5-expressing FCR3 and 3D7 parasites. Quantitative real-time PCR analyses showed that antibody selection resulted in a 3D7 line transcribing PF11_0008 (3D7-DC5) and in an FCR3 line transcribing IT4var02 (FCR3-DC5) at high levels (Figure 3). Flow cytometry assays with live parasites showed that most of the parasites in these lines expressed the native DC5-containing PfEMP1 on the surface of the infected erythrocytes, and confocal microscopy confirmed that the staining pattern was similar to that previously published for PfEMP1 [56,57] (Figure 4B). Interestingly, IgG against the DBLδ5-CIDRβ4 proteins were cross-reactive since the antibody reagents stained native DC5-PfEMP1 on the surface of erythrocytes infected with both the FCR3-DC5 and the 3D7-DC5 parasite line (Figure 4B). The antibodies did not react with parasites expressing the DC4-containing PFD1235w (Figure 4B), parasites expressing DC3-PfEMP1 [58] (unpublished data) or parasites expressing VAR2CSA (DC2-PfEMP1) (unpublished data). Cross-reactive anti-DC5 antibodies were not induced in all immunised animals. Out of 12 immunised rats, cross-reactive antibodies were induced in 4/6 animals immunised with DC5 DBLδ5-CIDRβ4 from FCR3 and 2/6 animals immunised with the recombinant construct from 3D7. Surface reactive antibodies reacting with the homologous DC5-PfEMP1 were induced in all animals ().

**Table 1:** Surface staining of FCR3 IT4var02 and 3D7 PF11_0008 with rat antibodies targeting DC5 domains.

**Gene**	**Rat antibody domain target**	**Positive antibody staining**	
		FCR3 IT4var02	3D7 PF11_0008
PF11_0008	DBLγ12	ND	ND
PF11_0008	DBLδ5	0/6	6/6
PF11_0008	CIDRβ4	**1/6**	4/6
PF11_0008	DBLβ9	0/6	3/6
PF11_0008	DBLδ5-CIDRβ4	**2/6**	6/6
IT4var02	DBLγ12	5/6	0/6
IT4var02	DBLδ5	6/6	0/6
IT4var02	CIDRβ4	6/6	0/6
IT4var02	DBLβ9	0/6	0/6
IT4var02	DBLδ5-CIDRβ4	6/6	**4/6**
IT4var02	DBLγ12-DBLδ5-CIDRβ4-DBLβ9	3/3	0/3

Cross-reactive antibodies are in bold.

To further evaluate the ability of DC5 domains to induce cross-reactive DC5 antibodies and to establish whether all DC5 domains were surface exposed on the native DC5-PfEMP1, we produced the DC5-PfEMP1 single domains from FCR3 and 3D7 and immunized rats (Table 1) and rabbits. The domain-specific IgG reacted with the homologous DC5-PfEMP1, but only one of them was DC5-PfEMP1 cross-reactive as recombinant CIDRβ4 of PF11_0008 (3D7) induced IgG that cross-reacted with native DC5-PfEMP1 on FCR3 in one of six immunized rats. Antibodies against a recombinant protein encompassing the entire four-domain DC5-PfEMP1 cassette (DBLγ12-DBLδ5-CIDRβ4-DBLβ9) from FCR3 also reacted with the native protein on FCR3-DC5 infected erythrocytes, but not with the DC5-PfEMP1 expressed by the 3D7-DC5 line (Table 1). Data obtained from immunization of rabbits were roughly similar to those obtained with rats (unpublished data). These experiments showed that the four domains constituting DC5 are all exposed on the surface of both FCR3 and 3D7 parasite lines expressing DC5-PfEMP1. IgG against single domains or the four-domain DC5 structure reacted with the homologues native protein, but in general not with heterologous DC5-PfEMP1. However, the two DBLδ5-CIDRβ4 recombinant proteins representing the central part of DC5 elicited surface- and cross-reactive antibodies.

### FCR3 IT4var02 binds PECAM1 expressed on TrHBMEC and binding is inhibited by anti-DC5 IgG

**Figure 5:**
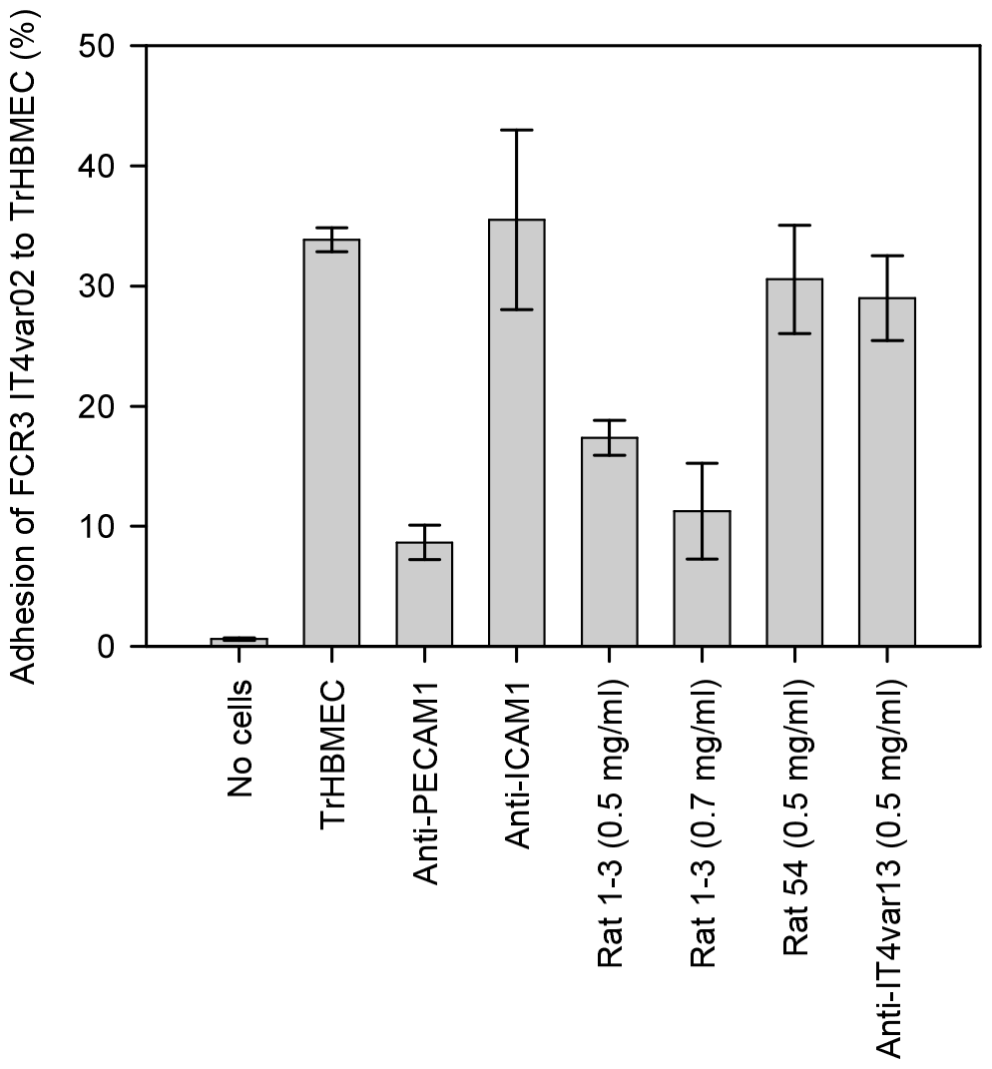
Adhesion of FCR3 parasites expressing IT4var02 (FCR3-DC5) to endothelial cells (TrHBMEC). Parasites were labeled with radioactivity and the y-axis shows the amount of radioactivity bound to the endothelial cells in percentage of the total amount of radioactivity added to the well. Parasites were tested after incubation with anti-PECAM1, anti-ICAM1, antibodies raised against the four-domain cassette 5 of IT4var02 (Rat 1-3), antibodies raised against a double-domain (DBLδ5-CIDRβ4) in IT4var02 DC5 (Rat 54) and control antibodies against a recombinant protein corresponding to full-length IT4var13. Error-bars are standard deviations, and the results are one representative experiment of three binding assays, except for inhibition with Rat 1-3, which was tested once.

We have previously shown that 3D7 parasites expressing PF11_0008 bind PECAM1 [57], and this binding phenotype was confirmed using the 3D7 PF11_0008 parasite line employed in the current study (unpublished data). It was not known whether this binding phenotype was mediated by sequences in the DC5 cassette present in PF11_0008 or some of the N-terminal PF11_0008 domains. To investigate another PfEMP1 containing the DC5-cassette, we tested the binding phenotype of the FCR3 parasite line expressing IT4var02 (FCR3-DC5). FCR3-DC5 bound strongly to Transformed Human Bone Marrow Endothelial Cells (TrHBMEC) and the binding was inhibited by antibodies to PECAM1, but not by antibodies to ICAM1 (Figure 5). These ICAM1 antibodies inhibit binding of FCR3 IT4var13 to CHO-cells expressing ICAM1 (unpublished data). The PECAM1 antibodies did not affect ICAM1 binding of a PFD1235w expressing 3D7 line (3D7-DC4) (unpublished data). Furthermore, IgG-purified rat antibodies elicited to the four-domain DC5-PfEMP1 cassette (DBLγ12-DBLδ5-CIDRβ4-DBLβ9) from IT4var02 that surface-stained the infected erythrocytes (Figure 4A) also inhibited binding (Figure 5). The cross-reactive IgG targeting DBLδ5-CIDRβ4 of FCR3 or 3D7 did not inhibit binding, and neither did control IgG purified from serum targeting another full-length recombinant PfEMP1 (IT4var13) known to bind ICAM1 (Figure 5) [59]. These anti-IT4var13 antibodies inhibit the ICAM1 binding of FCR3 parasites expressing IT4var13 (unpublished data).

### DC5-expressing infected erythrocytes are well recognized by plasma IgG from children living in an area of high malaria endemicity

**Figure 6:**
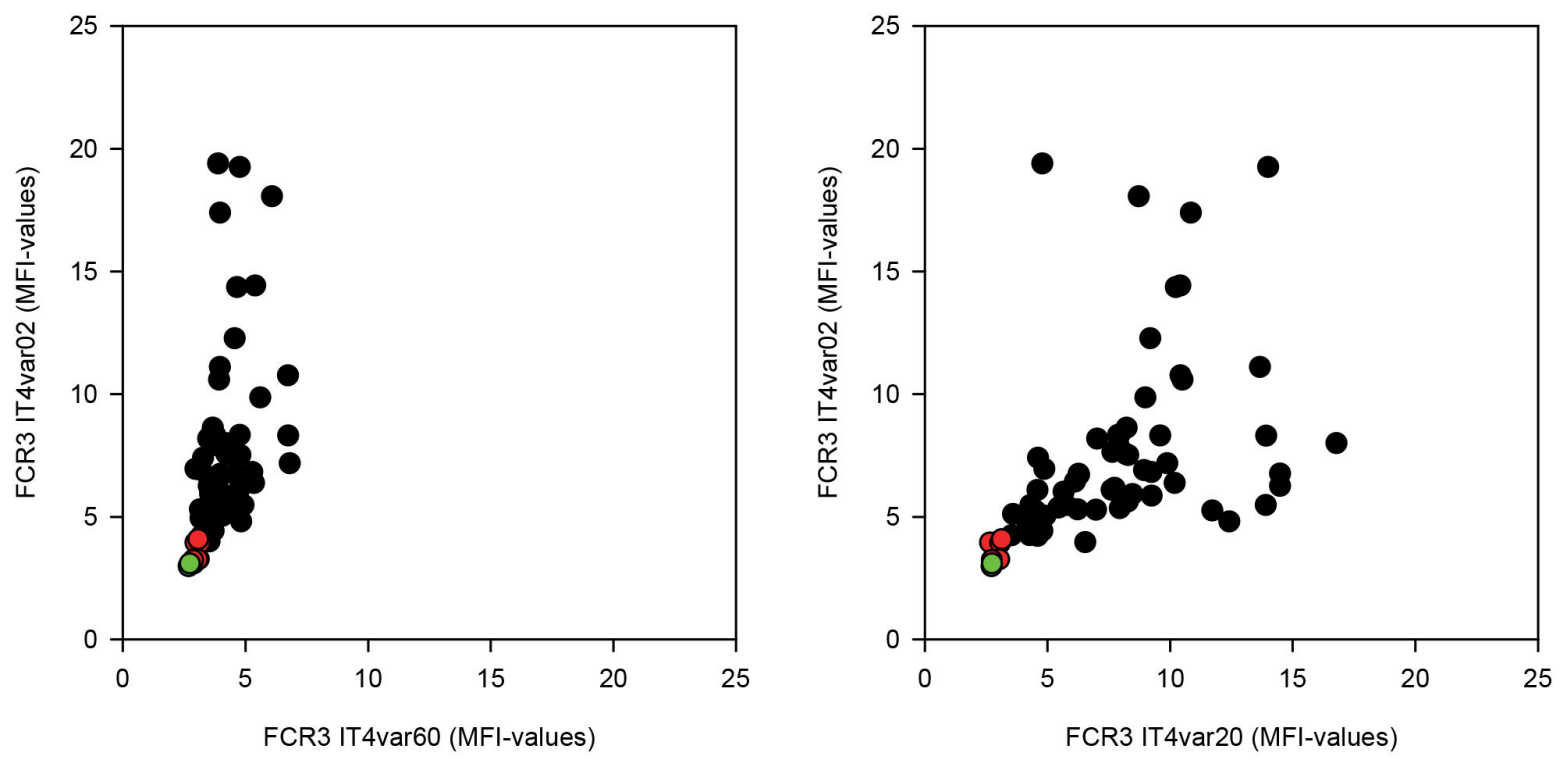
DC5-PfEMP1 expressing parasites are commonly recognised by serum from young Tanzanian children. Recognition by serum samples from Tanzanian children living in a holo-endemic area (black dots), Danish serum samples (red dots) and no serum (green dots). Recognition of FCR3 IT4var02 is compared to recognition of FCR3 ITseq60 and FCR3 IT4var20.

PfEMP1 surface-expressed by parasites causing severe malaria are more readily recognised by IgG in plasma collected from young children in malaria-endemic areas than PfEMP1 expressed by parasites causing uncomplicated disease [16,21]. To test the recognition of FCR3 parasites expressing IT4var02 (FCR3-DC5), we compared the ability of plasma antibodies collected from Tanzanian children to react with different PfEMP1 expressed on the surface on infected erythrocytes. The results showed that antibody reactivity was higher to infected erythrocytes expressing the DC5-containing IT4var02 than to those expressing IT4var60, a PfEMP1 mediating rosetting [60]. Antibodies reacted at similar levels to infected erythrocytes expressing either DC5-containing IT4var02 or IT4var20 (Figure 6), a PfEMP1 containing the DC8 associated with severe malaria [37,38,61].

### Plasma anti-DC5 IgG levels are associated with protection from malaria and hemoglobin level

We have previously shown that children acquire IgG to DC5 domains early in life [19,23,40]. For this study, we measured plasma antibody levels to 32 DBL-domains representing different DBL subgroups, including DBLγ12, DBLδ5 and the DBLβ9 present in the DC5 of PF11_0008, in 544 Tanzanian individuals aged two months to 60 years. The individuals were bled twice and monitored clinically during the six months between blood samplings. As reported previously, antibodies to DC5 domains were acquired early in life (unpublished and Figure S2) and logistic regression models showed that a log increase in antibody level in the sample collected before the monitoring was associated with a statistically significant reduction of suffering from malaria during the subsequent six-month period for all of the three DC5 domains and 18 of 29 other DBL-domains (Table 2).

**Table 2:** Association between clinical outcomes and plasma levels of antibodies to three DC5-PfEMP1 DBL domains and 29 DBL domains not belonging to DC5.

**Recombinant protein**	**Malaria fever risk**	**Hemoglobin**	**Hemoglobin 6 months after**
(native protein annotation)	Odds ratio[95% CI]	Regress. coeff. [95% CI]	Regress. coeff. [95% CI]
	(P value)	(P value)	(P value)
DBLγ12 (PF11_0008 DC5)	0.61 [0.41-0.94]	0.42 [0.13-0.71]	0.45 [0.18-0.73]
	(0.006)	(0.005)	(0.001)
DBLδ5 (PF11_0008 DC5)	0.43 [0.23-0.81]	n.s.	0.42 [0.06-0.67]
	(0.009)		(0.024)
DBLβ9 (PF13_0003 DC5)	0.49 [0.27-0.90]	n.s.	n.s.
	(0.021)		
Other DBL domains*:			
# associated**	18	1	2
Odds rt/regress. coeff.	0.01-0.69	1.42 [0.44-2.41]	0.50-0.54
P value	0.01-0.046	0.005	0.016-0.022

Antibody levels were measured in samples collected in April 2004 from 544 individuals living in two Tanzanian villages. Logistic regression was used to evaluate the risk of febrile malaria during the following six months and linear regression was used to predict hemoglobin levels (g/dl) at sampling and six months later. The models were corrected for age, sex, use of impregnated bed nets and home village. *29 additional DBL domains were tested, and are annotated in [19]. **Number of the 29 domains that were significantly associated in model.

Potential associations between hemoglobin levels and antibody levels were investigated by linear regression (Table 2). A log increase in antibody level against DC5-DBLγ12 at the time of the first bleeding was associated with an increase in hemoglobin level of 0.42 g/dl [0.13-0.71], and an increase of 0.45 g/dl [0.18-0.73] at the bleeding six month later. Anti-DBLδ5 DC5 levels were also associated with an increased hemoglobin level of 0.42 g/dl [0.06-0.67] at the bleeding six months after the antibody measurement. Only one and two of the 29 other anti-PfEMP1 levels measured showed association with hemoglobin level at the time of antibody measurement and six month later, respectively (Table 2). Antibody levels against MSP1-19 and MSP1-43 were not associated with protection from malaria or hemoglobin levels (unpublished data).

## Discussion

The severity of *P. falciparum* malaria infections is linked to the PfEMP1 expressed on the surface on infected erythrocytes. The type of PfEMP1 expressed also determines the phenotype of the parasite with respect to which receptors it binds on the vascular lining and serological recognition of the infected erythrocytes by plasma from malaria-exposed individuals. Parasites expressing the PfEMP1 type involved in pregnancy malaria, VAR2CSA bind Chondroitin Sulphate A (CSA) [30] and native VAR2CSA only react with IgG induced in women who have suffered from placental malaria. Acquisition of anti-VAR2CSA antibodies is associated with protection from placental malaria [31], and this has raised hope that it is possible to develop a VAR2CSA based vaccine to protect women from placental malaria [62].

Similarly, evidence indicates that antibodies against other PfEMP1 molecules are important mediators of the immunity acquired by children in endemic areas. Immunity is first acquired against severe and life-threatening infections, and later children develop a more broad protection against uncomplicated febrile malaria episodes [63]. This is thought to be caused by anti-PfEMP1 antibodies acquired in a non-random systematic way [19,23,64]. It has therefore been proposed that the anti-PfEMP1 antibodies developed during the first malaria infections target epitopes expressed on the surface of infected erythrocytes that have the potential to cause severe malaria infections. Thus, it is important to identify the PfEMP1 molecules that harbour domains targeted by antibodies acquired during the first malaria infections in young children, and to characterise the phenotype of parasites expressing these PfEMP1 types.

In 2004, we reported that 3D7 parasites expressing PF11_0008 had a serological phenotype corresponding to the phenotype of parasites causing severe malaria in young African children [20]. It was later shown that PF11_0008 DBL-domains were among the first and best-recognised DBL-domains [19,23] and that the presence of antibodies against the second CIDR-domain of PF11_0008 was associated with a reduced risk of developing febrile malaria [40]. Thus, anti-PF11_0008 antibodies are prevalent in people exposed to *P. falciparum* who cannot be assumed to have been exposed to 3D7 parasites expressing PF11_0008, but rather to parasites expressing serologically cross-reactive PfEMP1 molecules. PfEMP1 domains can be subgrouped according to their sequence similarity, and by analyses of the complete PfEMP1 repertoire of seven parasites, Rask et al. [29] identified a stretch of four domains of specific subtypes coded in PfEMP1 molecules in six of the seven genomes, including PF11_0008 of 3D7. This run of domains was classified as domain cassette 5 (DC5), and it must be assumed to be phylogenetically old as the structure was also found in the genome of the chimpanzee malaria parasite, *P* *. reichenowi* . In this paper, we have characterised DC5 further and compared the phenotype of two genetically distinct DC5-PfEMP1 parasite lines.

DC5 is a four-domain C-terminal cassette found exclusively among group A PfEMP1 and it is present in nearly all genomes investigated. The sequence homology within the DC5 domain subtypes is 50-60%, corresponding to the sequence homology between the other DCs, except for DC1-3, which are more conserved. The phylogenetic classification of PfEMP1 domains leading to the identification of the PfEMP1 domain cassettes represents an approximation of the sequence diversity over the entire length of the domains and therefore sequence traits common to DC5 and DC5-like homologues can be found. DBL domains are composed of three structural elements called sub-domain S1-3, whereas CIDR domains are composed of M1 and M2 sub-domains. While DBL and CIDR domains separate into distinct sub-types α-ζ and α-δ, the evolutionary history of the contained sub-domains can differ within a domain type and be shared with another. For example, the S3 of DBLα is most closely related to S3 of DBLδ, whereas the S2 of DBLα is most closely related to S2 of DBLβ [29].

The subdomain analysis of DC5 sequences and the sequence LOGO (Figure S1) indicate that the sequences that typify DC5 are spread throughout the cassette, but that sequences in the DBLγ subdomain 3 region and the adjacent DBLδ subdomain 1 region are particularly conserved and characteristic for DC5. In addition, there is a tendency that the domain subtype defining sequences are concentrated in the C-terminal subdomains (subdomain 3 of DBL domains and M2 for the CIDR domain). Interestingly, the sequence of subdomain 3 seems to determine the ICAM1 binding of the DBLβ PFD1235w [65]. We used DC5 antibodies to generate parasite lines expressing native DC5-PfEMP1 on the surface of infected erythrocytes and tested the reactivity of these lines to antibodies raised against each of the domains constituting DC5 in the native PfEMP1s. The results indicate that all of the four domains constituting DC5 are accessible to antibodies, when PfEMP1 harbouring DC5 are expressed on the surface of infected erythrocytes. This is also the case for the domains in VAR2CSA [66,67] and for the domains in DC4 [65], and it seems that even though domains interact to form a compact structure [10,68] few domains, if any, are completely buried in the native PfEMP1 structure, and they could in theory all contribute to binding. Antibodies generated against recombinant proteins representing single DC5 domains were generally not cross-reactive. This could reflect that the sequence homology is relatively low, typically 50-60%, which is lower than it is between corresponding domains in VAR2CSA and VAR3 to which cross-reactive antibodies are more readily induced [58,69,70]. However, we were able to induce cross-reactive antibodies with the two constructs that spanned the two central domains of DC5. This raises hope that it is possible to design antigenic constructs which induce antibodies with a broad DC specificity, a notion first supported by the fact that surface-reactive strain-transcending antibodies towards DBLα1.5 and DBLα1.8 domains of the DC16 can be induced [71]. Recently, cross-reactive antibodies to DC4 were also induced [65].

The relevance of the DC classification in terms of receptor binding capabilities is reliably determined for the binding of VAR2CSA to CSA, but also recently for the binding of DC4-PfEMP1 to ICAM1. We have previously found that the 3D7 parasite expressing the DC5 containing PF11_0008 binds endothelial cells via PECAM1 [57]. It was not clear whether the DC5 domains played a role in conveying this binding phenotype. In this study, our finding that FCR3 parasites expressing DC5-containing PfEMP1 (IT4var02) also bind PECAM1 and that this binding can be inhibited by DC5-specific antibodies support the notion that DC5 conveys PECAM1 binding. We found that binding was inhibited by IgG targeting the four-domain DC5-PfEMP1 cassette (DBLγ12-DBLδ5-CIDRβ4-DBLβ9) and not by the cross-reactive IgG targeting DBLδ5-CIDRβ4. However, cross-reactive antibodies are not necessarily inhibitory as previously shown with anti-VAR2CSA DBL5 antibodies, which often show cross-reactivity against native proteins expressed on different genetic backgrounds in flow cytometry assays but do not inhibit binding [72].

The PECAM1 binding ability of each of the recombinant DC5 proteins presented here was tested in ELISA, but although PECAM1 binding was observed for both the PF11_0008 and IT4var02 DBLβ9 domains and the PF11_0008 DBLδ5 domain, binding was not observed for the multi-domain constructs containing these domains nor was it possible to acquire sound binding kinetics data using surface plasmon resonance assays for these interactions (data not shown). It has previously been reported that recombinant CIDRα3.1 and DBLδ1 of IT4var21 bind PECAM1 in ELISA [73], but in that study, the PfEMP1:PECAM1 interaction did not reach saturation in titration curves. An FCR3 parasite line, which was thought to express IT4var21 also showed some PECAM1 binding [73], but later studies suggested that the parasite line employed (FCR3S1.2) did not express IT4var21 [60]. Thus, while the PECAM1 interaction of parasites does appear to be mediated through PfEMP1 it is unclear if this interaction is mediated by specific sub-domains of DBL domains or if indeed the interaction is dependent on full-length PfEMP1 molecules. PECAM1 is expressed by endothelial cells, monocytes, platelets and granulocytes [11] and parasite adhesion to this receptor appears to be common (50%) among field parasites [74]. If PfEMP1 containing DC5 bind PECAM1, this does not rule out that other PfEMP1 domains or structures could convey parasites this binding phenotype.

A previous study showed that antibodies to PF11_0008 correlated with protection from malaria infection and fever [40], and the results presented here also associate DC5 antibody levels to protection from malaria fever. Of the 32 cassettes included in the study, 3/3 DC5 constructs associated with protection from fever. However, this phenomenon was not exclusive for DC5, since 21 of the 32 tested domains showed this association. High levels of anti-DC5 antibodies were associated with an increase in blood hemoglobin levels among individuals exposed to malaria. A statistically significant association between hemoglobin levels was found for two of the three DC5 domains tested and only for two of the 29 other PfEMP1 domains tested. These findings suggest that DC5 antibodies play a role as effectors of malaria immunity, but do not answer whether DC5-PfEMP1-expressing parasites are commonly involved in precipitating severe malaria in young children. It was recently reported that parasites from children with severe malaria had high DC8 and DC13 transcript levels [61]. In some, but not the majority of these children there was also evidence of high levels of DC5 transcripts, and our current hypothesis is that DC5-expressing parasites are not the most important precipitators of severe malaria, but that parasites expressing DC5-PfEMP1 will occasionally cause severe infections.

In conclusion, by inducing antibodies towards DC5 that are cross-reactive with PfEMP1 containing the heterologous cassette, we have shown the biological relevance of subdividing consecutive PfEMP1 domains into DCs. We have established an adhesion phenotype that links DC5 surface expression to PECAM1 binding and have observed an association between anti-DC5 antibody levels and protection from malaria fever and anemia.

## Supporting Information

Figure S1 Sequence LOGO of DC5 based on 12 full-length DC5 sequences. The DBL and CIDR domains and sub-domain of the cassette are shown below the LOGO. The locations of HB331 and HB585, unique to DC5, are indicated by frames.
(TIF)

Figure S2 Age-stratified plasma anti-DBLγ12 PF11_0008 antibody levels. Antibody levels were analysed among 544 inhabitants living in Mkokola village (high malaria transmission) or Kwamasimba village (low malaria transmission).
(TIF)

Figure S3 SDS gel and western blots for recombinant DC5 proteins. (A) Table showing DC5 domains used for immunization and how they were added to gels. (B) SDS gel: Six µg per lane using BenchMark SDS markers. (C) Western blot incubated with anti-V5-HRP antibody (1:3000, Invitrogen).
(TIF)

Table S1 Primer target sites for cloning of recombinant PfEMP1 proteins. (TIF)

## References

[1] Murray CJ, Rosenfeld LC, Lim SS, Andrews KG, Foreman KJ et al. (2012) Global malaria mortality between 1980 and 2010: a systematic analysis. Lancet 379: 413-431. doi:10.1016/S0140-6736(12)60034-8. PubMed: 22305225. 22305225 10.1016/S0140-6736(12)60034-8

[2] World Health Organization (2011) World malaria Report 2011, Geneva Switzerland.

[3] Baruch DI, Pasloske BL, Singh HB, Bi X, Ma XC et al. (1995) Cloning the *P. falciparum* gene encoding PfEMP1, a malarial variant antigen and adherence receptor on the surface of parasitized human erythrocytes. Cell 82: 77-87. doi:10.1016/0092-8674(95)90054-3. PubMed: 7541722. 7541722 10.1016/0092-8674(95)90054-3

[4] Smith JD, Chitnis CE, Craig AG, Roberts DJ, Hudson-Taylor DE et al. (1995) Switches in expression of *Plasmodium falciparum var* genes correlate with changes in antigenic and cytoadherent phenotypes of infected erythrocytes. Cell 82: 101-110. doi:10.1016/0092-8674(95)90056-X. PubMed: 7606775. 7606775 10.1016/0092-8674(95)90056-x PMC3730239

[5] Su XZ, Heatwole VM, Wertheimer SP, Guinet F, Herrfeldt JA et al. (1995) The large diverse gene family *var* encodes proteins involved in cytoadherence and antigenic variation of *Plasmodium falciparum*-infected erythrocytes. Cell 82: 89-100. doi:10.1016/0092-8674(95)90055-1. PubMed: 7606788. 7606788 10.1016/0092-8674(95)90055-1

[6] Fried M, Duffy PE (1996) Adherence of *Plasmodium falciparum* to chondroitin sulfate A in the human placenta. Science 272: 1502-1504. doi:10.1126/science.272.5267.1502. PubMed: 8633247. 8633247 10.1126/science.272.5267.1502

[7] Treutiger CJ, Heddini A, Fernandez V, Muller WA, Wahlgren M (1997) PECAM-1/CD31, an endothelial receptor for binding *Plasmodium falciparum*-infected erythrocytes. Nat Med 3: 1405-1408. doi:10.1038/nm1297-1405. PubMed: 9396614. 9396614 10.1038/nm1297-1405

[8] Berendt AR, Simmons DL, Tansey J, Newbold CI, Marsh K (1989) Intercellular adhesion molecule-1 is an endothelial cell adhesion receptor for *Plasmodium falciparum* . Nature 341: 57-59. doi:10.1038/341057a0. PubMed: 2475784. 2475784 10.1038/341057a0

[9] Barnwell JW, Asch AS, Nachman RL, Yamaya M, Aikawa M et al. (1989) A human 88-kD membrane glycoprotein (CD36) functions *in vitro* as a receptor for a cytoadherence ligand on *Plasmodium falciparum*-infected erythrocytes. J Clin Invest 84: 765-772. doi:10.1172/JCI114234. PubMed: 2474574. 2474574 10.1172/JCI114234 PMC329717

[10] Clausen TM, Christoffersen S, Dahlbäck M, Langkilde AE, Jensen KE et al. (2012) Structural and functional insight into how the Plasmodium falciparum VAR2CSA protein mediates binding to chondroitin sulfate A in placental malaria. J Biol Chem 287: 23332-23345. doi:10.1074/jbc.M112.348839. PubMed: 22570492. 22570492 10.1074/jbc.M112.348839 PMC3390611

[11] Rowe JA, Claessens A, Corrigan RA, Arman M (2009) Adhesion of *Plasmodium falciparum*-infected erythrocytes to human cells: molecular mechanisms and therapeutic implications. Expert Rev Mol Med 11: e16. doi:10.1017/S1462399409001082. PubMed: 19467172. 19467172 10.1017/S1462399409001082 PMC2878476

[12] Hviid L (2010) The role of *Plasmodium falciparum* variant surface antigens in protective immunity and vaccine development. Hum Vaccin 6: 84-89. doi:10.4161/hv.6.1.9602. PubMed: 19823032. 19823032 10.4161/hv.6.1.9602

[13] Gupta S, Snow RW, Donnelly CA, Marsh K, Newbold C (1999) Immunity to non-cerebral severe malaria is acquired after one or two infections. Nat Med 5: 340-343. doi:10.1038/6560. PubMed: 10086393. 10086393 10.1038/6560

[14] Bull PC, Lowe BS, Kortok M, Molyneux CS, Newbold CI et al. (1998) Parasite antigens on the infected red cell surface are targets for naturally acquired immunity to malaria. Nat Med 4: 358-360. doi:10.1038/nm0398-358. PubMed: 9500614. 9500614 10.1038/nm0398-358 PMC3836255

[15] Bull PC, Lowe BS, Kortok M, Marsh K (1999) Antibody recognition of *Plasmodium falciparum* erythrocyte surface antigens in Kenya: evidence for rare and prevalent variants. Infect Immun 67: 733-739. PubMed: 9916084. 9916084 10.1128/iai.67.2.733-739.1999 PMC96380

[16] Bull PC, Kortok M, Kai O, Ndungu F, Ross A et al. (2000) *Plasmodium falciparum*-infected erythrocytes: agglutination by diverse Kenyan plasma is associated with severe disease and young host age. J Infect Dis 182: 252-259. doi:10.1086/315652. PubMed: 10882604. 10882604 10.1086/315652

[17] Bull PC, Marsh K (2002) The role of antibodies to *Plasmodium falciparum*-infected-erythrocyte surface antigens in naturally acquired immunity to malaria. Trends Microbiol 10: 55-58. doi:10.1016/S0966-842X(01)02278-8. PubMed: 11827798. 11827798 10.1016/s0966-842x(01)02278-8

[18] Bull PC, Berriman M, Kyes S, Quail MA, Hall N et al. (2005) *Plasmodium falciparum* variant surface antigen expression patterns during malaria. PLOS Pathog 1: e26. doi:10.1371/journal.ppat.0010026. PubMed: 16304608. 16304608 10.1371/journal.ppat.0010026 PMC1287908

[19] Cham GK, Turner L, Lusingu J, Vestergaard L, Mmbando BP et al. (2009) Sequential, ordered acquisition of antibodies to *Plasmodium falciparum* erythrocyte membrane protein 1 domains. J Immunol 183: 3356-3363. doi:10.4049/jimmunol.0901331. PubMed: 19675168. 19675168 10.4049/jimmunol.0901331

[20] Jensen AT, Magistrado P, Sharp S, Joergensen L, Lavstsen T et al. (2004) *Plasmodium falciparum* associated with severe childhood malaria preferentially expresses PfEMP1 encoded by group A *var* genes. J Exp Med 199: 1179-1190. doi:10.1084/jem.20040274. PubMed: 15123742. 15123742 10.1084/jem.20040274 PMC2211911

[21] Nielsen MA, Staalsoe T, Kurtzhals JA, Goka BQ, Dodoo D et al. (2002) *Plasmodium falciparum* variant surface antigen expression varies between isolates causing severe and nonsevere malaria and is modified by acquired immunity. J Immunol 168: 3444-3450. PubMed: 11907103. 11907103 10.4049/jimmunol.168.7.3444

[22] Bull PC, Buckee CO, Kyes S, Kortok MM, Thathy V et al. (2008) *Plasmodium falciparum* antigenic variation. Mapping mosaic *var* gene sequences onto a network of shared, highly polymorphic sequence blocks. Mol Microbiol 68: 1519-1534. doi:10.1111/j.1365-2958.2008.06248.x. PubMed: 18433451. 18433451 10.1111/j.1365-2958.2008.06248.x PMC2440560

[23] Cham GK, Turner L, Kurtis JD, Mutabingwa T, Fried M et al. (2010) Hierarchical, domain type-specific acquisition of antibodies to *Plasmodium falciparum* erythrocyte membrane protein 1 in Tanzanian children. Infect Immun 78: 4653-4659. doi:10.1128/IAI.00593-10. PubMed: 20823214. 20823214 10.1128/IAI.00593-10 PMC2976311

[24] Kalmbach Y, Rottmann M, Kombila M, Kremsner PG, Beck HP et al. (2010) Differential *var* gene expression in children with malaria and antidromic effects on host gene expression. J Infect Dis 202: 313-317. doi:10.1086/653586. PubMed: 20540611. 20540611 10.1086/653586

[25] Kyriacou HM, Stone GN, Challis RJ, Raza A, Lyke KE et al. (2006) Differential *var* gene transcription in *Plasmodium falciparum* isolates from patients with cerebral malaria compared to hyperparasitaemia. Mol Biochem Parasitol 150: 211-218. doi:10.1016/j.molbiopara.2006.08.005. PubMed: 16996149. 16996149 10.1016/j.molbiopara.2006.08.005 PMC2176080

[26] Rottmann M, Lavstsen T, Mugasa JP, Kaestli M, Jensen AT et al. (2006) Differential expression of *var* gene groups is associated with morbidity caused by *Plasmodium falciparum* infection in Tanzanian children. Infect Immun 74: 3904-3911. doi:10.1128/IAI.02073-05. PubMed: 16790763. 16790763 10.1128/IAI.02073-05 PMC1489729

[27] Warimwe GM, Keane TM, Fegan G, Musyoki JN, Newton CR et al. (2009) *Plasmodium falciparum var* gene expression is modified by host immunity. Proc Natl Acad Sci U S A 106: 21801-21806. doi:10.1073/pnas.0907590106. PubMed: 20018734. 20018734 10.1073/pnas.0907590106 PMC2792160

[28] Lavstsen T, Salanti A, Jensen AT, Arnot DE, Theander TG (2003) Sub-grouping of *Plasmodium falciparum* 3D7 *var* genes based on sequence analysis of coding and non-coding regions. Malar J 2: 27. doi:10.1186/1475-2875-2-27. PubMed: 14565852. 14565852 10.1186/1475-2875-2-27 PMC222925

[29] Rask TS, Hansen DA, Theander TG, Gorm PA, Lavstsen T (2010) Plasmodium falciparum erythrocyte membrane protein 1 diversity in seven genomes--divide and conquer. PLOS Comput Biol 6: e1000933 PubMed: 20862303. 20862303 10.1371/journal.pcbi.1000933 PMC2940729

[30] Salanti A, Staalsoe T, Lavstsen T, Jensen AT, Sowa MP et al. (2003) Selective upregulation of a single distinctly structured *var* gene in chondroitin sulphate A-adhering *Plasmodium falciparum* involved in pregnancy-associated malaria. Mol Microbiol 49: 179-191. doi:10.1046/j.1365-2958.2003.03570.x. PubMed: 12823820. 12823820 10.1046/j.1365-2958.2003.03570.x

[31] Salanti A, Dahlbäck M, Turner L, Nielsen MA, Barfod L et al. (2004) Evidence for the involvement of VAR2CSA in pregnancy-associated malaria. J Exp Med 200: 1197-1203. doi:10.1084/jem.20041579. PubMed: 15520249. 15520249 10.1084/jem.20041579 PMC2211857

[32] Magistrado P, Salanti A, Tuikue Ndam NG, Mwakalinga SB, Resende M et al. (2008) VAR2CSA expression on the surface of placenta-derived *Plasmodium falciparum*-infected erythrocytes. J Infect Dis 198: 1071-1074. doi:10.1086/591502. PubMed: 18700835. 18700835 10.1086/591502

[33] Bian Z, Wang G (2000) Antigenic *var*iation and cytoadherence of PfEMP1 of *Plasmodium falciparum*-infected erythrocyte from malaria patients. Chin Med J (Engl) 113: 981-984. PubMed: 11776131. 11776131

[34] Kirchgatter K, Portillo HA (2002) Association of severe noncerebral *Plasmodium falciparum* malaria in Brazil with expressed PfEMP1 DBL1 alpha sequences lacking cysteine residues. Mol Med 8: 16-23. PubMed: 11984002. 11984002 PMC2039937

[35] Falk N, Kaestli M, Qi W, Ott M, Baea K et al. (2009) Analysis of *Plasmodium falciparum var* genes expressed in children from Papua New Guinea. J Infect Dis 200: 347-356. doi:10.1086/600071. PubMed: 19552523. 19552523 10.1086/600071

[36] Kaestli M, Cockburn IA, Cortés A, Baea K, Rowe JA et al. (2006) Virulence of malaria is associated with differential expression of *Plasmodium falciparum var* gene subgroups in a case-control study. J Infect Dis 193: 1567-1574. doi:10.1086/503776. PubMed: 16652286. 16652286 10.1086/503776 PMC2877257

[37] Avril M, Tripathi AK, Brazier AJ, Andisi C, Janes JH et al. (2012) A restricted subset of *var* genes mediates adherence of *Plasmodium falciparum*-infected erythrocytes to brain endothelial cells. Proc Natl Acad Sci U S A 109: E1782-E1790. doi:10.1073/pnas.1120534109. PubMed: 22619321. 22619321 10.1073/pnas.1120534109 PMC3387091

[38] Claessens A, Adams Y, Ghumra A, Lindergard G, Buchan CC et al. (2012) A subset of group A-like *var* genes encodes the malaria parasite ligands for binding to human brain endothelial cells. Proc Natl Acad Sci U S A 109: E1772-E1781. doi:10.1073/pnas.1120992109. PubMed: 22619330. 22619330 10.1073/pnas.1120461109 PMC3387129

[39] Lavstsen T, Magistrado P, Hermsen CC, Salanti A, Jensen AT et al. (2005) Expression of *Plasmodium falciparum* erythrocyte membrane protein 1 in experimentally infected humans. Malar J 4: 21. doi:10.1186/1475-2875-4-21. PubMed: 15857512. 15857512 10.1186/1475-2875-4-21 PMC1112614

[40] Magistrado PA, Lusingu J, Vestergaard LS, Lemnge M, Lavstsen T et al. (2007) Immunoglobulin G antibody reactivity to a group A *Plasmodium falciparum* erythrocyte membrane protein 1 and protection from *P. falciparum* malaria. Infect Immun 75: 2415-2420. doi:10.1128/IAI.00951-06. PubMed: 17283085. 17283085 10.1128/IAI.00951-06 PMC1865733

[41] Edgar RC (2004) MUSCLE: multiple sequence alignment with high accuracy and high throughput. Nucleic Acids Res 32: 1792-1797. doi:10.1093/nar/gkh340. PubMed: 15034147. 15034147 10.1093/nar/gkh340 PMC390337

[42] Hall TA (1999) Bioedit: a user-friendly biological sequence alignment editor and analysis program for Windows 95/98/NT. Nucleic Acids Symp Ser 41: 95-98.

[43] Tamura K, Dudley J, Nei M, Kumar S (2007) MEGA4: Molecular Evolutionary Genetics Analysis (MEGA) software version 4.0. Mol Biol Evol 24: 1596-1599. doi:10.1093/molbev/msm092. PubMed: 17488738. 17488738 10.1093/molbev/msm092

[44] Crooks GE, Hon G, Chandonia JM, Brenner SE (2004) WebLogo: a sequence logo generator. Genome Res 14: 1188-1190. doi:10.1101/gr.849004. PubMed: 15173120. 15173120 10.1101/gr.849004 PMC419797

[45] Cranmer SL, Magowan C, Liang J, Coppel RL, Cooke BM (1997) An alternative to serum for cultivation of *Plasmodium falciparum in vitro* . Trans R Soc Trop Med Hyg 91: 363-365. doi:10.1016/S0035-9203(97)90110-3. PubMed: 9231219. 9231219 10.1016/s0035-9203(97)90110-3

[46] Schweitzer KM, Vicart P, Delouis C, Paulin D, Dräger AM et al. (1997) Characterization of a newly established human bone marrow endothelial cell line: distinct adhesive properties for hematopoietic progenitors compared with human umbilical vein endothelial cells. Lab Invest 76: 25-36. PubMed: 9010447. 9010447

[47] Staalsoe T, Nielsen MA, Vestergaard LS, Jensen AT, Theander TG et al. (2003) *In vitro* selection of *Plasmodium falciparum* 3D7 for expression of variant surface antigens associated with severe malaria in African children. Parasite Immunol 25: 421-427. doi:10.1111/j.1365-3024.2003.00652.x. PubMed: 14651589. 14651589 10.1111/j.1365-3024.2003.00652.x

[48] Moll K, Ljungström I, Perlmann H, Scherf A, Wahlgren M (2009). Methods in Malar Res www MR 4 org/Portals/3/Methods_In_Malaria_Research-5theditionv5-2 pdf

[49] Dahlbäck M, Lavstsen T, Salanti A, Hviid L, Arnot DE et al. (2007) Changes in *var* gene mRNA levels during erythrocytic development in two phenotypically distinct *Plasmodium falciparum* parasites. Malar J 6: 78. doi:10.1186/1475-2875-6-78. PubMed: 17565661. 17565661 10.1186/1475-2875-6-78 PMC1904452

[50] Salanti A, Jensen AT, Zornig HD, Staalsoe T, Joergensen L et al. (2002) A sub-family of common and highly conserved *Plasmodium falciparum var* genes. Mol Biochem Parasitol 122: 111-115. doi:10.1016/S0166-6851(02)00080-4. PubMed: 12076777. 12076777 10.1016/s0166-6851(02)00080-4

[51] Sander AF, Salanti A, Lavstsen T, Nielsen MA, Magistrado P et al. (2009) Multiple VAR2CSA-type PfEMP1 genes located at different chromosomal loci occur in many *Plasmodium falciparum* isolates. PLOS ONE 4: e6667. doi:10.1371/journal.pone.0006667. PubMed: 19690615. 19690615 10.1371/journal.pone.0006667 PMC2723927

[52] Cham GK, Kurtis J, Lusingu J, Theander TG, Jensen AT et al. (2008) A semi-automated multiplex high-throughput assay for measuring IgG antibodies against *Plasmodium falciparum* erythrocyte membrane protein 1 (PfEMP1) domains in small volumes of plasma. Malar J 7: 108. doi:10.1186/1475-2875-7-108. PubMed: 18549480. 18549480 10.1186/1475-2875-7-108 PMC2435541

[53] Dahlbäck M, Jørgensen LM, Nielsen MA, Clausen TM, Ditlev SB et al. (2011) The chondroitin sulfate A-binding site of the VAR2CSA protein involves multiple N-terminal domains. J Biol Chem 286: 15908-15917. doi:10.1074/jbc.M110.191510. PubMed: 21398524. 21398524 10.1074/jbc.M110.191510 PMC3091200

[54] Mmbando BP, Vestergaard LS, Kitua AY, Lemnge MM, Theander TG et al. (2010) A progressive declining in the burden of malaria in north-eastern Tanzania. Malar J 9: 216. doi:10.1186/1475-2875-9-216. PubMed: 20650014. 20650014 10.1186/1475-2875-9-216 PMC2920289

[55] Mmbando BP, Lusingu JP, Vestergaard LS, Lemnge MM, Theander TG et al. (2009) Parasite threshold associated with clinical malaria in areas of different transmission intensities in north eastern Tanzania. BMC Med Res Methodol 9: 75. doi:10.1186/1471-2288-9-75. PubMed: 19909523. 19909523 10.1186/1471-2288-9-75 PMC2781814

[56] Bengtsson D, Sowa KM, Salanti A, Jensen AT, Joergensen L et al. (2008) A method for visualizing surface-exposed and internal PfEMP1 adhesion antigens in *Plasmodium falciparum* infected erythrocytes. Malar J 7: 101. doi:10.1186/1475-2875-7-101. PubMed: 18533996. 18533996 10.1186/1475-2875-7-101 PMC2453135

[57] Joergensen L, Bengtsson DC, Bengtsson A, Ronander E, Berger SS et al. (2010) Surface co-expression of two different PfEMP1 antigens on single *Plasmodium falciparum*-infected erythrocytes facilitates binding to ICAM1 and PECAM1. PLOS Pathog 6: e1001083 PubMed: 20824088. 20824088 10.1371/journal.ppat.1001083 PMC2932717

[58] Wang CW, Lavstsen T, Bengtsson DC, Magistrado PA, Berger SS et al. (2012) Evidence for *in vitro* and *in vivo* expression of the conserved *var*3 (type 3) *Plasmodium falciparum* erythrocyte membrane protein 1. Malar J 11: 129. doi:10.1186/1475-2875-11-129. PubMed: 22533832. 22533832 10.1186/1475-2875-11-129 PMC3407477

[59] Brown A, Turner L, Christoffersen S, Andrews KA, Szestak T et al. (2013) Molecular architecture of a complex between an adhesion protein from the malaria parasite and intracellular adhesion molecule 1. J Biol Chem 288: 5992-6003. doi:10.1074/jbc.M112.416347. PubMed: 23297413. 23297413 10.1074/jbc.M112.416347 PMC3581401

[60] Albrecht L, Moll K, Blomqvist K, Normark J, Chen Q et al. (2011) *var* gene transcription and PfEMP1 expression in the rosetting and cytoadhesive *Plasmodium falciparum* clone FCR3S1.2. Malar J 10: 17. doi:10.1186/1475-2875-10-17. PubMed: 21266056. 21266056 10.1186/1475-2875-10-17 PMC3036667

[61] Lavstsen T, Turner L, Saguti F, Magistrado P, Rask TS et al. (2012) *Plasmodium falciparum* erythrocyte membrane protein 1 domain cassettes 8 and 13 are associated with severe malaria in children. Proc Natl Acad Sci U S A 109: E1791-E1800. doi:10.1073/pnas.1120455109. PubMed: 22619319. 22619319 10.1073/pnas.1120455109 PMC3387094

[62] Hviid L (2011) The case for PfEMP1-based vaccines to protect pregnant women against *Plasmodium falciparum* malaria. Expert Rev Vaccines 10: 1405-1414. doi:10.1586/erv.11.113. PubMed: 21988306. 21988306 10.1586/erv.11.113

[63] Hviid L (2005) Naturally acquired immunity to *Plasmodium falciparum* malaria in Africa. Acta Trop 95: 270-275. doi:10.1016/j.actatropica.2005.06.012. PubMed: 16018958. 16018958 10.1016/j.actatropica.2005.06.012

[64] Joergensen L, Vestergaard LS, Turner L, Magistrado P, Lusingu JP et al. (2007) 3D7-Derived *Plasmodium falciparum* erythrocyte membrane protein 1 is a frequent target of naturally acquired antibodies recognizing protein domains in a particular pattern independent of malaria transmission intensity. J Immunol 178: 428-435. PubMed: 17182581. 17182581 10.4049/jimmunol.178.1.428

[65] Bengtsson A, Joergensen L, Rask TS, Olsen RW, Andersen MA et al. (2013) A novel domain cassette identifies Plasmodium falciparum PfEMP1 proteins binding ICAM-1 and is a target of cross-reactive, adhesion-inhibitory antibodies. J Immunol 190: 240-249. doi:10.4049/jimmunol.1202578. PubMed: 23209327. 23209327 10.4049/jimmunol.1202578 PMC3539686

[66] Barfod L, Nielsen MA, Turner L, Dahlbäck M, Jensen AT et al. (2006) Baculovirus-expressed constructs induce immunoglobulin G that recognizes VAR2CSA on *Plasmodium falciparum*-infected erythrocytes. Infect Immun 74: 4357-4360. doi:10.1128/IAI.01617-05. PubMed: 16790811. 16790811 10.1128/IAI.01617-05 PMC1489712

[67] Nielsen MA, Pinto VV, Resende M, Dahlbäck M, Ditlev SB et al. (2009) Induction of adhesion-inhibitory antibodies against placental *Plasmodium falciparum* parasites by using single domains of VAR2CSA. Infect Immun 77: 2482-2487. doi:10.1128/IAI.00159-09. PubMed: 19307213. 19307213 10.1128/IAI.00159-09 PMC2687338

[68] Vigan-Womas I, Guillotte M, Juillerat A, Hessel A, Raynal B et al. (2012) Structural basis for the ABO blood-group dependence of *Plasmodium falciparum* rosetting. PLOS Pathog 8: e1002781 PubMed: 22807674. 22807674 10.1371/journal.ppat.1002781 PMC3395597

[69] Bigey P, Gnidehou S, Doritchamou J, Quiviger M, Viwami F et al. (2011) The NTS-DBL2X region of VAR2CSA induces cross-reactive antibodies that inhibit adhesion of several *Plasmodium falciparum* isolates to chondroitin sulfate A. J Infect Dis 204: 1125-1133. doi:10.1093/infdis/jir499. PubMed: 21881129. 21881129 10.1093/infdis/jir499

[70] Barfod L, Dobrilovic T, Magistrado P, Khunrae P, Viwami F et al. (2010) Chondroitin sulfate A-adhering *Plasmodium falciparum*-infected erythrocytes express functionally important antibody epitopes shared by multiple variants. J Immunol 185: 7553-7561. doi:10.4049/jimmunol.1002390. PubMed: 21078904. 21078904 10.4049/jimmunol.1002390 PMC4785892

[71] Ghumra A, Semblat JP, Ataide R, Kifude C, Adams Y et al. (2012) Induction of strain-transcending antibodies against Group A PfEMP1 surface antigens from virulent malaria parasites. PLOS Pathog 8: e1002665. 22532802 10.1371/journal.ppat.1002665 PMC3330128

[72] Salanti A, Resende M, Ditlev SB, Pinto VV, Dahlbäck M et al. (2010) Several domains from VAR2CSA can induce Plasmodium falciparum adhesion-blocking antibodies. Malar J 9: 11. doi:10.1186/1475-2875-9-S2-O11. PubMed: 20064234. 20064234 10.1186/1475-2875-9-11 PMC2817698

[73] Chen Q, Heddini A, Barragan A, Fernandez V, Pearce SF et al. (2000) The semiconserved head structure of *Plasmodium falciparum* erythrocyte membrane protein 1 mediates binding to multiple independent host receptors. J Exp Med 192: 1-10. doi:10.1084/jem.192.1.1. PubMed: 10880521. 10880521 10.1084/jem.192.1.1 PMC1887712

[74] Heddini A, Chen Q, Obiero J, Kai O, Fernandez V et al. (2001) Binding of *Plasmodium falciparum*-infected erythrocytes to soluble platelet endothelial cell adhesion molecule-1 (PECAM-1/CD31): frequent recognition by clinical isolates. Am J Trop Med Hyg 65: 47-51. PubMed: 11504407. 11504407 10.4269/ajtmh.2001.65.47

