# Supplementary figures and images for: Plasmodium falciparum Expressing Domain Cassette 5 Type PfEMP1 (DC5-PfEMP1) Bind PECAM1
Source: PLoS One. 2013 Jul 9;8(7):e69117. doi: 10.1371/journal.pone.0069117 (PMC3706608; doi:10.1371/journal.pone.0069117)

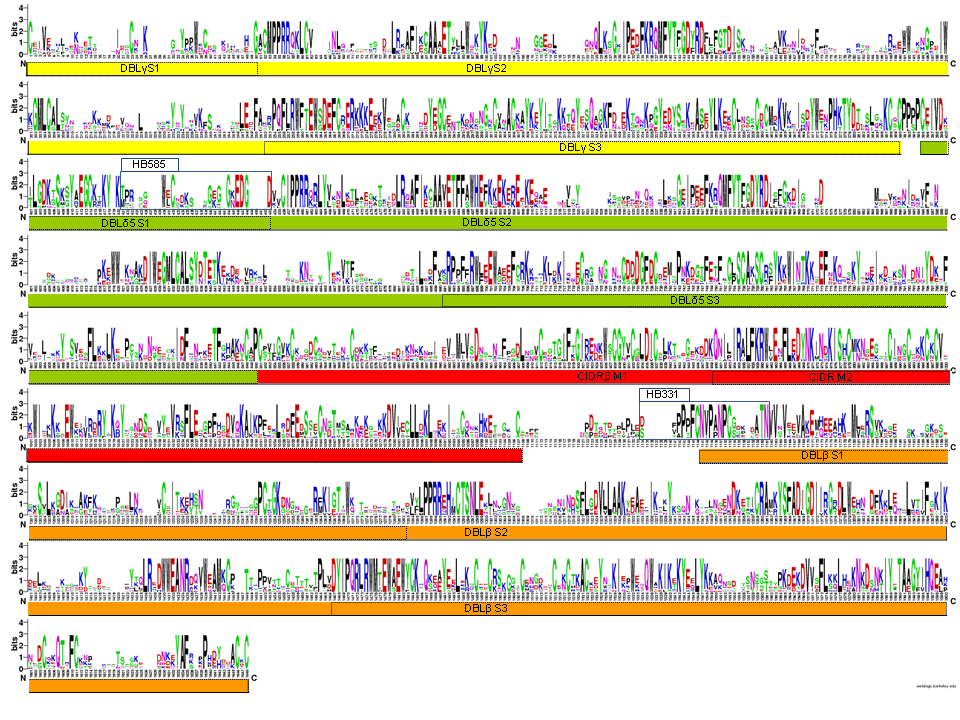

Supplement: Figure S1 — The DBL and CIDR domains and sub-domain of the cassette are shown below the LOGO. The locations of HB331 and HB585, unique to DC5, are indicated by frames. (TIF) [file pone.0069117.s001.tif]

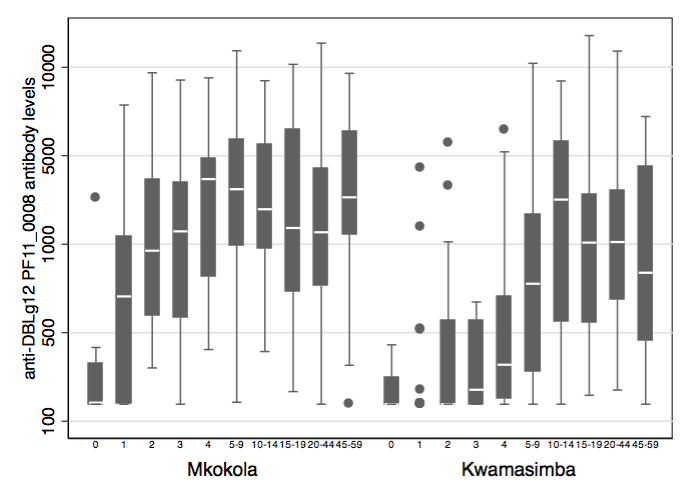

Supplement: Figure S2 — Antibody levels were analysed among 544 inhabitants living in Mkokola village (high malaria transmission) or Kwamasimba village (low malaria transmission). (TIF) [file pone.0069117.s002.tif]

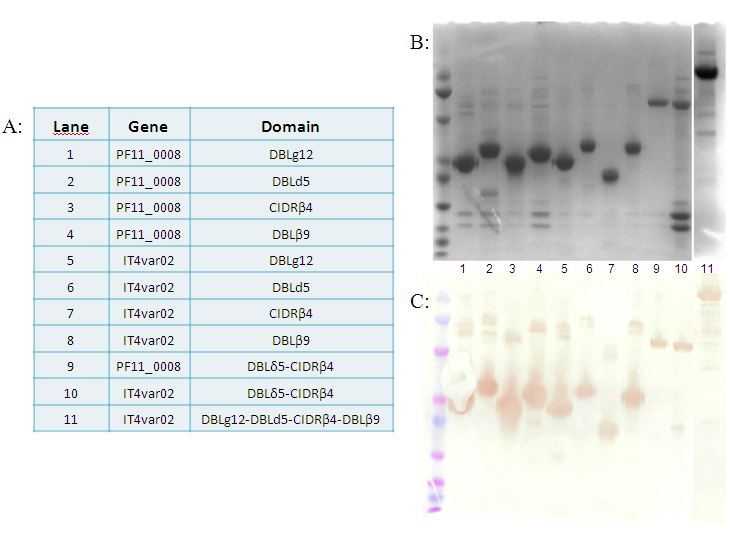

Supplement: Figure S3 — (A) Table showing DC5 domains used for immunization and how they were added to gels. (B) SDS gel: Six µg per lane using BenchMark SDS markers. (C) Western blot incubated with anti-V5-HRP antibody (1:3000, Invitrogen). (TIF) [file pone.0069117.s003.tif]

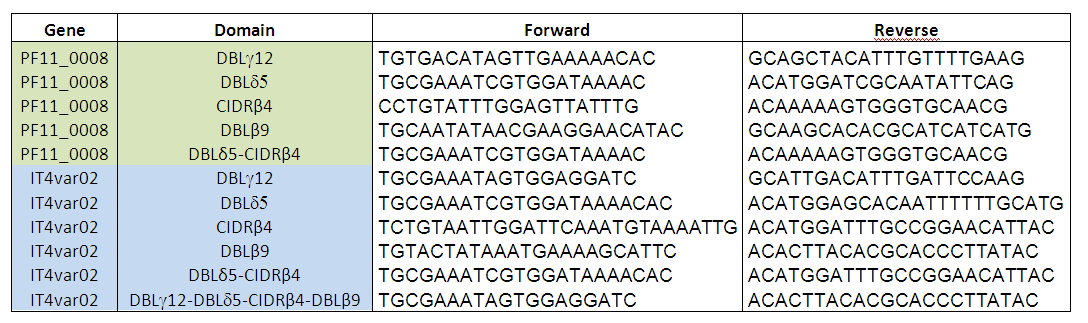

Supplement: Table S1 — (TIF) [file pone.0069117.s004.tif]
